# Does Climate Change Increase Crop Water Requirements of Winter Wheat and Summer Maize in the Lower Reaches of the Yellow River Basin?

**DOI:** 10.3390/ijerph192416640

**Published:** 2022-12-11

**Authors:** Kun Jia, Wei Zhang, Bingyan Xie, Xitong Xue, Feng Zhang, Dongrui Han

**Affiliations:** 1 School of Management Engineering, Shandong Jianzhu University, Jinan 250101, China; 2 Institute of Agricultural Information and Economics, Shandong Academy of Agricultural Sciences, Jinan 250100, China

**Keywords:** crop water requirements, spatial and temporal variation, climate change, sensitivity analysis, attribution analysis, Yellow River Basin

## Abstract

With increasing water resources stress under climate change, it is of great importance to deeply understand the spatio-temporal variation of crop water requirements and their response to climate change for achieving better water resources management and grain production. However, the quantitative evaluation of climate change impacts on crop water requirements and the identification of determining factors should be further explored to reveal the influencing mechanism and actual effects thoroughly. In this study, the water requirements of winter wheat and summer maize from 1981 to 2019 in the lower reaches of the Yellow River Basin were estimated based on the Penman–Monteith model and crop coefficient method using daily meteorological data. Combined with trends test, sensitivity and contribution analysis, the impacts of different meteorological factors on crop water requirement variation were explored, and the dominant factors were then identified. The results indicated that the temperature increased significantly (a significance level of 0.05 was considered), whereas the sunshine duration, relative humidity and wind speed decreased significantly from 1981 to 2019 in the study area. The total water requirements of winter wheat and summer maize presented a significant decreasing trend (−1.36 mm/a) from 1981 to 2019 with a multi-year average value of 936.7 mm. The crop water requirements of winter wheat was higher than that of summer maize, with multi-year average values of 546.6 mm and 390.1 mm, respectively. In terms of spatial distribution patterns, the crop water requirement in the north was generally higher than that in the south. The water requirements of winter wheat and summer maize were most sensitive to wind speed, and were less sensitive to the minimum temperature and relative humidity. Wind speed was the leading factor of crop water requirement variation with the highest contribution rate of 116.26% among the considered meteorological factors. The results of this study will provide important support for strengthening the capacity to cope with climate change and realizing sustainable utilization of agricultural water resources in the lower reaches of the Yellow River Basin.

## 1. Introduction

Agricultural water consumption accounts for more than 70% of total water consumption worldwide, which restricts the social economy development and is directly related to food security [1,2]. According to the Sixth Assessment Report released by the Intergovernmental Panel on Climate Change (IPCC), global warming has become an undisputed fact and the trend is expected to continue, as there has been an increasing mean temperature since 1880 [3]. Studies have shown that climate change has a considerable impact on global water cycles, as well as on agriculture, and the sustainable use of agricultural water resources is facing continuing challenges, especially in regions with unbalanced water supply and demand [4,5,6,7].

As a key indicator of determining agricultural water utilization schedules, crop water requirements (CWR) are vulnerable to climate change. Exploring the spatio-temporal variation and conducting climatic attribution analysis of CWR variation is of great significance for understanding the impact mechanism of climate change and developing adaptation strategies. At present, numerous studies have been carried out and the contents have mainly been concentrated on the historical spatio-temporal variations, influencing factors and future simulation in water requirements of different crops [8,9,10,11]. For example, Wu et al. calculated the irrigation water requirements and analyzed the spatio-temporal variations from 1980 to 2012 of the North China Plain [12]. Ruan et al. estimated CWR in the Syr Darya Basin of Central Asia and investigated the dominant factors of CWR variations [13]. Yang et al. projected the future irrigation water requirement under two different climate scenarios in the Lower Mississippi Alluvial Valley [14].

The response of crop water requirements to climate change in different regions has attracted much attention in recent years [15,16,17,18]. Yang et al. examined CWR variation trends and revealed the impacts of climate change on cotton water requirements from 1965 to 2016 on the North China Plain [19]. Xu et al. evaluated the impacts of climate change on wheat water requirements from 1960 to 2019 in the Beijing-Tianjin-Hebei region in China [20]. Ding et al. assessed the effects of climate change on rice water requirements in the middle and lower reaches of the Yangtze River [21]. Crops such as wheat, maize and rice were always chosen as the study object because of their importance in regional food security. However, the thorough comparison of climate change impacts on different crops in the same region is still insufficient, especially in the winter wheat-summer maize rotation system. The quantitative evaluation of climate change impacts on CWR and the dominant influencing factors should be further explored. In addition, many methods, such as the correlation analyses, could describe the relationship between climate change and CWR [22], and the combination of sensitivity and contribution analysis is meaningful to reveal the mechanism and actual effects of climate change on CWR.

The Yellow River is the second longest river in China, and the basin is an important economic and ecological zone. The agriculture sector is the largest user of water resources, especially in the lower reaches of the Yellow River Basin (LYRB), which is a vital grain production area of China [23]. Due to the shortage of water resources and large crop water requirements, a series of ecological and environmental problems, such as soil erosion, have been caused [24]. Meanwhile, climate change has been reported to have important impact on water resources in the LYRB [25,26]. Therefore, it is imperative to achieve rational agricultural water use under climate change for sustainable development of the LYRB. Meanwhile, as the main grain producing area and climate change sensitive area, related studies in the LYRB mainly focused on the estimation of crop water consumption or the impacts of climate change on some parameters such as evapotranspiration and runoff [27,28].

In this study, the CWR variation of winter wheat and summer maize in LYRB was analyzed and the impacts of climate change were then evaluated. The objectives of this study were to (1) estimate the CWR of winter wheat and summer maize and analyze the spatio-temporal variation from 1981 to 2019 in LYRB; (2) explore the sensitivity of the CWR variations to different meteorological factors; (3) investigate the contribution of meteorological factors to CWR variations and identify the dominant factors for better understanding the impacts of climate change. The results will provide valuable information for effective agricultural water utilization and sustainable water management in LYRB.

## 2. Materials and Methods

### 2.1. Study Area

**Figure 1:**
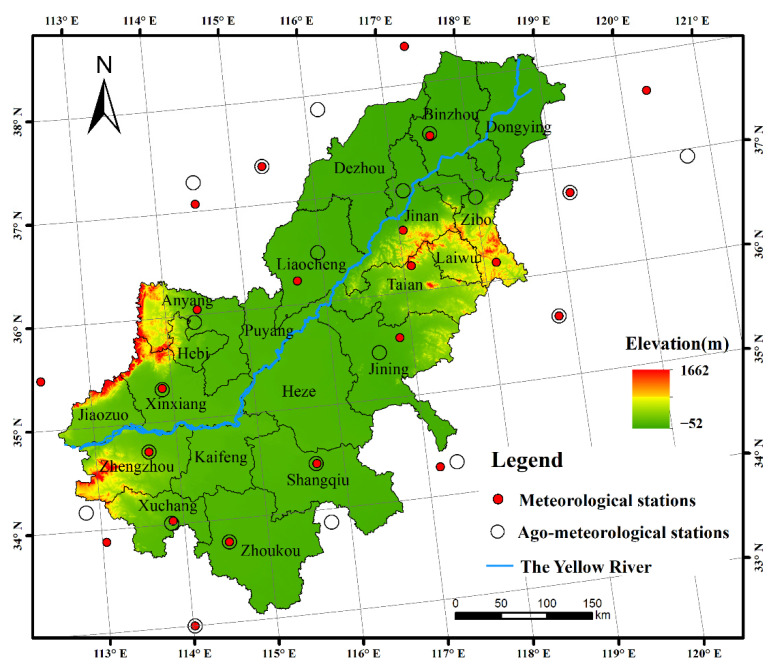
Study area.

Located in the eastern part of China, the LYRB ranges from the latitudes of 33° N to 39° N and the longitudes of 112° E to 120° E, mainly covering areas of Shandong and Henan province (Figure 1). The region is dominated by a temperate monsoon climate and the terrain mainly includes plains, mountains and hills, with a wide distribution of cultivated land. The winter wheat and summer maize double cropping system is the major cropping pattern in the LYRB. In general, winter wheat in the LYRB is sown in early October and harvested in early or mid-June of the following year, and the main growth period of summer maize is from the end of May to September.

### 2.2. Datasets

Daily meteorological data from 1981 to 2019 and agrometeorological data were collected from the China Meteorological Administration (http://data.cma.cn). The observed meteorological data mainly includes the maximum temperature, minimum temperature, sunshine duration, relative humidity and wind speed. The agrometeorological data mainly includes the crop phenological phase and crop parameters, and the multi-year average values were adopted in this study.

### 2.3. Methods

#### 2.3.1. Estimation of Crop Water Requirements

According to the method recommended by the Food and Agriculture Organization (FAO), the CWR could be calculated by multiplying the reference evapotranspiration by the crop coefficient [29]. The formula is defined as follows:

(1) $$ET_{c}=ET_{0}\times Kc$$

where *ET_c_* is the CWR (mm), *ET*_0_ is the reference evapotranspiration (mm) and *K_c_* is the crop coefficient. *ET*_0_ is calculated using the Penman–Monteith equation as follows [29]:

(2) $$ET_{0}=\frac{0.408\Delta (R_{n}-G)+\gamma \frac{900}{T+273}u_{2}(e_{s}-e_{a})}{\Delta +\gamma (1+0.34u_{2})}$$

where *R_n_* is the net radiation at the land surface (MJ/(m^2^·d)), *G* is the soil heat flux density (MJ/(m^2^·d)), *T* is the mean air temperature at a height of 2 m (°C), *u*_2_ is the wind speed at a height of 2 m (m/s), *e_s_* is the saturation vapor pressure (kPa), *e_a_* is the actual vapor pressure (kPa), *γ* is the psychrometric constant (kPa/°C) and Δ is the slope of vapor pressure curve (kPa/°C).

The crop growing periods can be separated into four distinct stages: initial, crop development, mid-season and late season. The recommended *K_c_* for each stage was adjusted according to the local condition, and the daily *K_c_* was calculated. The formula is defined as follows [29]:

(3) $$K_{c}=K_{ctab}+[0.04(u_{2}-2)-0.004(RH_{\min}-45)]{\left(\frac{h}{3}\right)}^{0.3}$$

where *K_ctab_* is the recommended crop coefficients at different periods under certain meteorological conditions (*RH_min_* ≈ 45%, *u_2_* ≈ 2 m/s), *RH_min_* is the minimum relative humidity (%) and *h* is crop height (m).

#### 2.3.2. Trends Analysis

The Mann–Kendall test and Sen’s slope were adopted to analyze the variation trend of meteorological factors and CWR, and this method has been widely used in hydrological and meteorological time series studies [30,31,32]. The values of Z statistic indicate trends and significance, and the values of Slope statistic indicate the trend rate. The formula is defined as follows:

(4) $$Z=\left\{ \begin{array}{cc} \frac{S\;-\;1}{\sqrt{\mathrm{var}\left(S\right)}}, & S>0\\ 0, & S=0\\ \frac{S\;-\;1}{\sqrt{\mathrm{var}\left(S\right)}}, & S>0 \end{array}\right.$$

(5) $$S=\sum_{i=1}^{n-1}\sum_{k=i+1}^{n}\mathrm{sgn}\left(x_{k}-x_{i}\right)$$

(6) $$\mathrm{sgn}\left(\theta \right)=\left\{ \begin{array}{ll} 1, & \theta >0\\ 0, & \theta =0\\ -1, & \theta <0 \end{array}\right.$$

(7) var[S]=[n(n−1)(2n+5)−∑t(t−1)(2t+5)]/18

where *x_k_* and *x_i_* represent the sequential values, *t* represents the extent of any given tie and *n* is the length of the time series.

(8) $$\mathrm{Slope}=\mathrm{median}\left(\frac{x_{i}-x_{j}}{i-j}\right)$$

where 1 < *j* < *i* < *n*, and the slope is considered as the median of the entire dataset.

#### 2.3.3. Sensitivity and Contribution Analysis

Sensitivity analysis can quantitatively assess the impact of meteorological factors on the change of CWR [33,34,35]. The dimensionless relative sensitivity coefficient was used in this study, and the formula is defined as follows:

(9) $$Sv_{i}=\underset{\Delta v_{i}\to 0}{\lim}(\frac{\Delta ET_{c}/ET_{c}}{\Delta v_{i}/v_{i}})=\frac{\partial ET_{c}}{\partial v_{i}}\cdot \frac{v_{i}}{ET_{c}}$$

where Δ*v_i_* is the relative change in meteorological factor, and Δ*ET_c_* is relative change in CWR induced by Δ*v_i_*. The positive (negative) sensitivity coefficient represents the CWR variation and is consistent with (or contrary to) the meteorological factor changes. The higher sensitivity coefficient indicates the greater impact of meteorological factors on the CWR variation.

The relative change of meteorological factors multiplied by the related sensitivity coefficient could indicate the actual contribution of one factor to the CWR change [36,37,38]. The formula is defined as follows:

(10) $$Cv_{i}=\frac{\Delta v_{i}}{\left|{\bar{v}}_{i}\right|}\cdot Sv_{i}$$

where *Cv_i_* represents the contribution of the meteorological factor to the CWR change, ${\bar{v}}_{i}$ is the mean value of the meteorological factor and the Δ*v_i_* is the relative change of meteorological factors, which is obtained by multiplying the slope value by the length of study period. The actual CWR change could be approximately equal to the sum of the relative changes caused by all meteorological factors, and the proportion of each meteorological factor contribution represents the contribution rate.

## 3. Results

### 3.1. Variation in Meteorological Factors

The variation characteristics of meteorological factors from 1981 to 2019 in the LYRB are shown in Table 1. Under the background of global climate change, an obvious warming trend has also been found in the LYRB from 1981 to 2019. The annual maximum and minimum temperatures exhibited significant (a significance level of 0.05 was considered in this study) increasing trends of 0.03 °C/a and 0.05 °C/a, respectively, with multi-year average values of 19.13 °C and 9.08 °C, respectively. The annual sunshine duration showed a significant decreasing trend of −0.02 h/a with a mean value of 6.07 h. The annual relative humidity and wind speed both decreased significantly with multi-year average values of 65.69% and 2.52 m/s, respectively.

### 3.2. Spatial and Temporal Variation of CWR

#### 3.2.1. Temporal Variation

**Figure 2:**
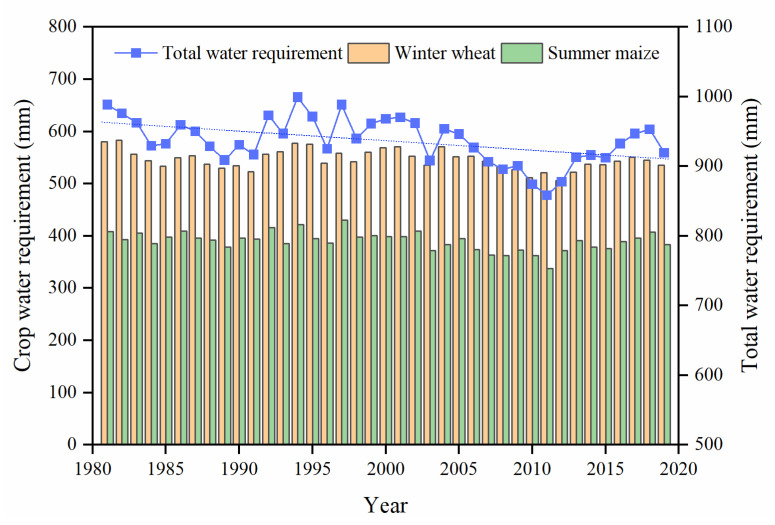
Variation of crop water requirement of winter wheat and summer maize from 1981 to 2019 in the lower reaches of the Yellow River Basin.

The total annual CWR of winter wheat and summer maize presented a significant decreasing trend of −1.36 mm/a in general from 1981 to 2019 in the LYRB (Figure 2). The multi-year average value of total CWR was 936.7 mm, with the highest CWR of 999.4 mm in 1994 and the lowest CWR of 858.3 mm in 2011. In different time periods, the multi-year average value of total CWR from 1981 to 2000 was 952.9 mm, whereas the CWR decreased slightly in general from 2001 to 2019 with a multi-year average value of 919.6 mm. From the perspective of different crop types, the CWR variation of winter wheat and summer maize was similar with significant decreasing trends of −0.63 mm/a and −0.65 mm/a, respectively. The CWR of winter wheat was higher than that of summer maize, and the multi-year average values were 546.6 mm and 390.1 mm, respectively.

#### 3.2.2. Spatial Pattern

**Figure 3:**
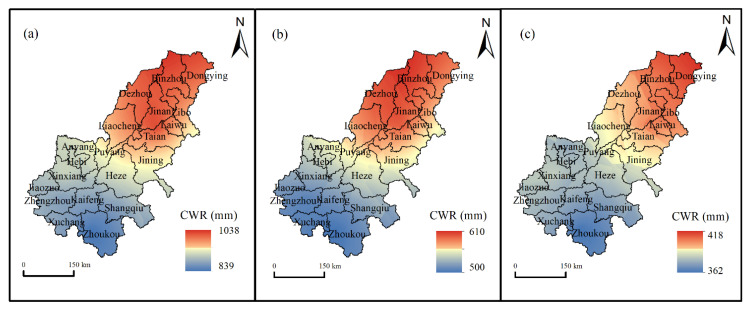
Spatial distribution of multi-year average crop water requirement of winter wheat and summer maize from 1981 to 2019 in the lower reaches of the Yellow River Basin. (**a**–**c**) Toal water requirement, winter wheat, and summer maize, respectively.

The spatial pattern of multi-year average CWR of winter wheat and summer maize from 1981 to 2019 in the LYRB is shown in Figure 3. Generally, the total CWR gradually increased from the southwest to the northeast of the study area, ranging from 839 mm to 1038 mm (Figure 3a). The total CWR in Binzhou, Dongying, Jinan and Dezhou were relatively high (>1000 mm), whereas the total CWR in Zhoukou, Xuchang, Kaifeng and Shangqiu were relatively low (<900 mm). As shown in Figure 3b,c, the CWR of winter wheat and summer maize were also high in the north and low in the south, and the spatial pattern was relatively consistent with total CWR.

### 3.3. Sensitivity of CWR to Meteorological Factors Change

**Figure 4:**
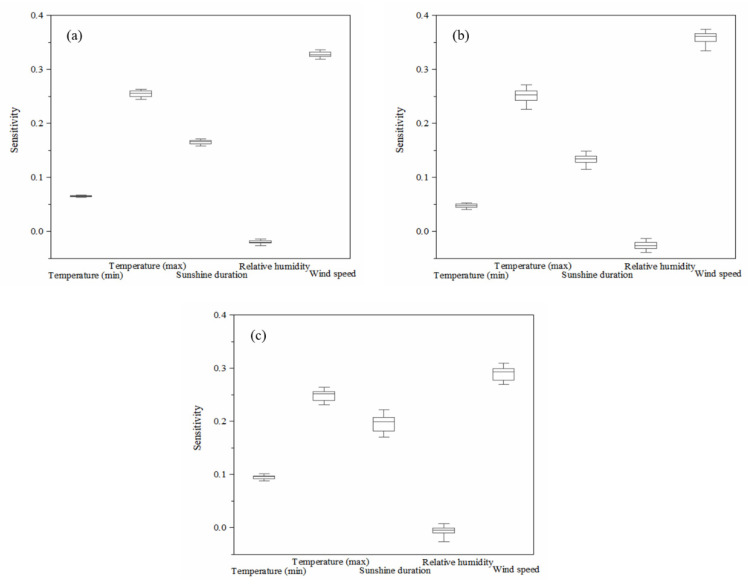
Sensitivity of crop water requirement of winter wheat and summer maize to meteorological factors change in the lower reaches of the Yellow River Basin. (**a**–**c**) Toal water requirement, winter wheat, and summer maize, respectively.

The sensitivity of CWR to different meteorological factors was quantified to better understand the response of CWR variations to climate change. As shown in Figure 4a, the highest sensitivity of total CWR was to wind speed with a value of 0.33, indicating that if the wind speed increased (decreased) by 10% (with other factors remaining unchanged), the total CWR of winter wheat and summer maize would also increase (decrease) by 3.3%. The sensitivity of total CWR to maximum temperature was also high, with a sensitivity coefficient of 0.25, whereas the sensitivity coefficient to minimum temperature was only 0.07, indicating that total CWR variation was more susceptible to changes in maximum temperature than minimum temperature. The sensitivity of total CWR to sunshine duration was slightly lower than that of maximum temperature, with a sensitivity coefficient of 0.16. The sensitivity coefficient of total CWR to relative humidity was −0.02, indicating that an increase in relative humidity will lead to a decrease in CWR. The impacts of minimum temperature and relative humidity on CWR may be limited due to the low sensitivity coefficient.

As for winter wheat and summer maize (Figure 4b,c), the sensitivities of CWR to wind speed were also the highest, with sensitivity coefficients of 0.36 and 0.29, respectively. The CWR sensitivity coefficients of winter wheat and summer maize to maximum temperature were both 0.25, whereas CWR sensitivity of summer maize to minimum temperature was higher than that of winter wheat. In terms of sunshine duration, the CWR sensitivity coefficients of winter wheat and summer maize were 0.13 and 0.20, respectively. Particularly, the sensitivities of CWR of both winter wheat and summer maize to relative humidity were generally low.

### 3.4. Contributions of Meteorological Factors to CWR Variation

**Figure 5:**
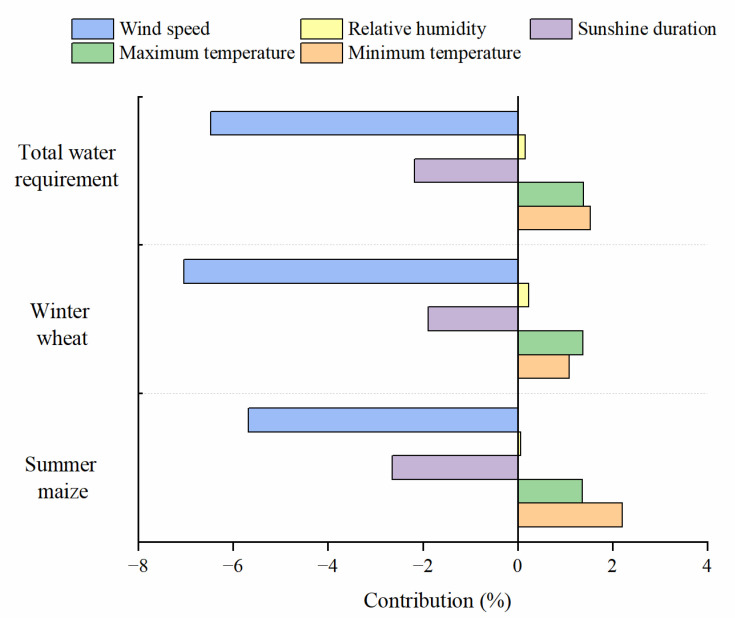
Contribution of meteorological factors to crop water requirement variation in the lower reaches of the Yellow River Basin.

Combining the sensitivity coefficient and variation trends, the actual contribution of meteorological factors to CWR variations were explored. The results showed that wind speed was the dominant factor influencing the CWR variations of winter wheat and summer maize in the LYRB from 1981 to 2019 (Figure 5). The contribution of wind speed to total CWR variation of winter wheat and summer maize was −6.47%, indicating that the reduction in wind speed led to a 6.47% decrease in total CWR. Meanwhile, the wind speed contributed more to CWR variation of winter wheat than that of summer maize. The contribution of temperature increase to the variation of total CWR was 2.92%, and the maximum and minimum temperature contributed 1.39% and 1.53%, respectively. Through the relatively high sensitivity of CWR to maximum temperature, the contribution of minimum temperature to total CWR variation was generally higher than the maximum temperature because of the larger relative change. The reduction in sunshine duration resulted a decrease in CWR, and the contributions were −2.18%, −1.88% and −2.64% in total, winter wheat and summer maize CWR variations, respectively. The decrease of relative humidity led to an increase of CWR, and the contribution was the lowest in the CWR variations.

**Figure 6:**
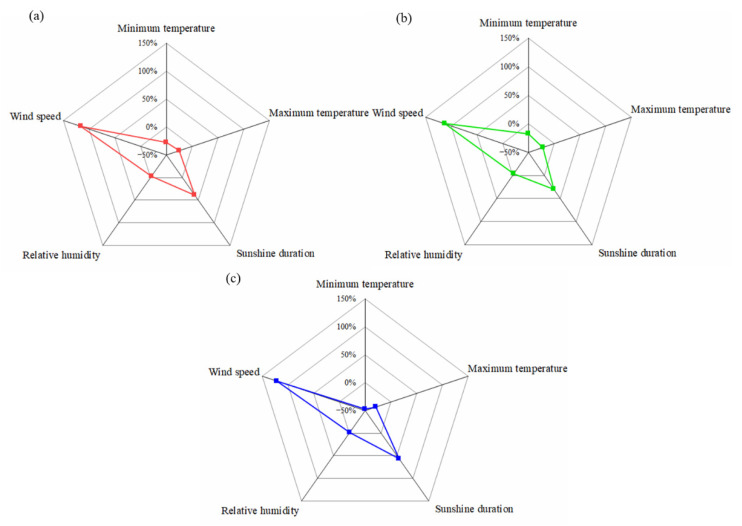
Contribution rate of meteorological factors to crop water requirement variation in the lower reaches of the Yellow River Basin. (**a**–**c**) Toal water requirement, winter wheat, and summer maize, respectively.

Concerning the contribution rate of meteorological factors to CWR variation of winter wheat and summer maize in the LYRB from 1981 to 2019 (Figure 6a), wind speed had the highest contribution rate of 116.26% to the total CWR variation, followed by sunshine durations with a contribution rate of 39.21%. Because of the opposite variation trends, the contribution rates of minimum temperature and maximum temperature to the total CWR variation were −27.49% and −25.02%, respectively. Although the increasing temperature will result in an increase in CWR, the CWR still decreased significantly. Therefore, the effect of increasing temperature on CWR variation may be offset by impacts caused by other meteorological factors. The contribution rate of relative humidity was only −2.96%. Similar contribution characteristics of meteorological factors to CWR variation in winter wheat and summer maize are presented in Figure 6b,c. It is worth noting that the contribution rates of minimum temperature to CWR variation in winter wheat and summer maize were −17.53 and −47.18%, respectively. The contribution rate of sunshine duration to CWR variation in summer maize reached 56.42%, which was 26.22% higher than that of winter wheat.

## 4. Discussion

The CWR of winter wheat and summer maize presented a significant decreasing trend from 1981 to 2019 in the LYRB, and this finding was relatively consistent with the results of previous studies [12]. However, Wang et al. found that the irrigation water requirements of winter wheat showed no prominent trend from 2000 to 2020 in the North China Plain [15]. In fact, the variation of CWR exhibited certain spatio-temporal variability, which was deeply affected by study periods and region. Liu et al. also showed that the water requirement variation showed obvious spatial differences in the regions of the Yellow River Basin [26]. Therefore, it is always important to pay attention to the spatio-temporal variation of CWR in scientific agricultural water planning.

Wind speed was deemed the dominant factor of CWR variation in our study because of the high sensitivity and substantial reduction from 1981 to 2019. The studies of Yin et al. also indicated that wind speed was the leading factor of potential evapotranspiration (a key parameter in estimating CWR) variation in northern China [33]. It is worth noting that the determining meteorological factors of CWR variation were not only influenced by regional climatic conditions but also by the complex mathematical relationships between CWR and factors. The sensitivity of CWR to maximum temperature showed a relatively high of 0.25, indicating that the CWR was also susceptible to maximum temperature change. Meanwhile, the impacts of temperature change on CWR variation should not be ignored, considering the absolute contribution rate of 52.51% (−27.49% of minimum temperature and −25.02% of maximum temperature) in the LYRB. Though the impact was offset to a certain degree in the study period by other meteorological factors, the increasing temperatures may continue to result in CWR increase and cause agricultural water pressure in the future. Therefore, assessing the impacts of climate change on CWR variation is meaningful for adopting effective strategies.

The CWR variation trends and response to climate change of winter wheat was found to be relatively similar to those of summer maize in the LYRB. However, the differences between different crops still need to be concerned for delicacy management. The CWR of winter wheat was generally higher than that of summer maize, indicating that winter wheat may play a more important role in regional agricultural water use than summer maize. In addition, the impacts of minimum temperature and sunshine duration on summer maize were greater than that on winter wheat. Considering the CWR characteristics of different crops and adjusting the cropping pattern or planting region may be helpful for rational agricultural water utilization [23].

Generally speaking, the impacts of climate change on CWR variation is complex, and many additional factors need to be considered, such as the changes in phenological period and planting region [39,40,41]. Meanwhile, meteorological factors may exist in complicated interactions, which will increase the uncertainty of the results. In future studies, taking various factors into consideration and adopting multiple methods on different spatio-temporal scales will improve the research accuracy and provide important reference to achieve sustainable agricultural water management. In addition, human activities and the induced change, such as land use and soil properties change, have been reported to exert great pressure on water resources [42,43,44,45]. With intensified human activities, it is imperative to conduct a comprehensive study of the impacts of climate change and human activities on regional crop water requirements in the future.

## 5. Conclusions

Based on the crop coefficient method, trend test, sensitivity and contribution analysis, the spatio-temporal variations of CWR for winter wheat and summer maize were explored, and the impacts of climate change on CWR variations were revealed from 1981 to 2019 in the LYRB. The results showed that the annual maximum and minimum temperature increased significantly, whereas sunshine duration, relative humidity and wind speed decreased significantly from 1981 to 2019. The total CWR of winter wheat and summer maize presented a significant decreasing trend (−1.36 mm/a) with a multi-year average value of 936.7 mm, and the CWR of winter wheat was higher than that of summer maize. The CWR of winter wheat and summer maize showed similar spatial distribution, and generally increased from the southwest to the northeast. The CWR of winter wheat and summer maize were highly sensitive to wind speed, followed by maximum temperature, sunshine duration, minimum temperature and relative humidity. Wind speed was the dominant factor of CWR variation and presented the greatest impacts with the highest contribution rate of 116.26%. Increasing temperatures also had a relatively great influence on CWR variation, whereas this impact was offset by other meteorological factors. The contribution of relative humidity to CWR variation was relatively limited. The results of this study will provide a scientific basis for mitigating the impacts of climate change and improving agricultural water management.

## Figures and Tables

**Table 1:** Variation characteristics of meteorological factors from 1981 to 2019 in the lower reaches of the Yellow River Basin. A significance level of 0.05 was considered.

Characteristics	Maximum Temperature (°C)	Minimum Temperature (°C)	Sunshine Duration (h)	Relative Humidity (%)	Wind Speed (m/s)
Trends	Significant increase	Significant increase	Significant decrease	Significant decrease	Significant decrease
Slope/year	0.03	0.05	−0.02	−0.14	−0.01
Mean	19.13	9.08	6.07	65.69	2.52

## Data Availability

The meteorological data were obtained from China Meteorological Administration (http://data.cma.cn/).
